# A small-molecule DS44170716 inhibits Ca^2+^-induced mitochondrial permeability transition

**DOI:** 10.1038/s41598-017-03651-7

**Published:** 2017-06-20

**Authors:** Naohiro Kon, Atsushi Satoh, Naoki Miyoshi

**Affiliations:** 1 Medical Science Department, Daiichi Sankyo Co., Ltd., Tokyo, Japan; 2 Manufacturing Department III, Kitasato Daiichi Sankyo Vaccine Co., Ltd., Saitama, Japan; 3 End-Organ Disease Laboratories, Daiichi Sankyo Co., Ltd., Tokyo, Japan

## Abstract

Mitochondria are involved in a variety of physiological and pathological processes. Ca^2+^ uptake is one of the important functions of the organelle for maintenance of cellular Ca^2+^ homeostasis. In pathological conditions such as ischemia reperfusion injury, Ca^2+^ overload into mitochondria induces mitochondrial permeability transition (MPT), a critical step for cell death. Because inhibition of MPT is a promising approach to protecting cells and organs, it is important for drug discovery to identify novel chemicals or mechanisms to inhibit MPT. Here we report upon a small-molecule compound DS44170716 that inhibits Ca^2+^-induced MPT in rat liver isolated mitochondria. DS44170716 protects human liver HepG2 cells from Ca^2+^-induced death with a level of protection similar to cyclosporin A (CsA). The inhibitory mechanism of DS44170716 against MPT is independent on PPIF, a target of CsA. DS44170716 blocks Ca^2+^ flux into the mitochondria by decreasing mitochondrial membrane potential, while potently inhibiting mitochondrial complex III activities and weakly inhibiting complex IV and V activities. Similarly, complex III inhibitor antimycin A, complex IV inhibitor KCN or complex V inhibitor oligomycin inhibits Ca^2+^ uptake of isolated mitochondria. These results show that DS44170716 is a novel class inhibitor of MPT by blocking of mitochondrial complexes and Ca^2+^-overload into mitochondria.

## Introduction

Mitochondria generate cellular energy by oxidative phosphorylation^1, 2^. Mitochondrial complexes I to IV produce a proton gradient across the inner membrane of mitochondria, and complex V converts ADP to ATP by using energy of the membrane potential. In addition to their role as a cellular energy source, mitochondria maintain cellular Ca^2+^ homeostasis by uptake of Ca^2+^ in response to cytosolic Ca^2+^ increase^3^. In a pathological condition, a massive amount of Ca^2+^ enters mitochondria and induces mitochondrial permeability transition (MPT)^4^. The MPT causes disruption of the mitochondrial membrane and releases cytochrome c, which triggers several necrotic or apoptotic cell death cascades.

Mitochondrial Ca^2+^ overload is involved in various pathological events, including ischemia-reperfusion injury^5^. Immunosuppressant cyclosporin A (CsA) has been shown to inhibit MPT and protect against cellular damage in several tissues^6, 7^. NIM-811, a non-immunosuppressive analog of CsA, also protects against ischemia-reperfusion injury^8–11^, showing that the mitochondrial action of CsA is critical for the protective effect. Sanglifehrin A and antamanide also inhibit MPT and cell death by inhibiting the same target, peptidyl-prolyl cis-trans isomerase F (PPIF)^12, 13^. PPIF-deficient mice show protective effects in a variety of pathological models in addition to that of reperfusion injury^14–18^. These results suggest that the development of an MPT inhibitor would be a promising approach to protecting cells and tissues.

Despite intensive studies in biochemistry and molecular biology, the mechanism of MPT remains unsolved^19^. Because atractyloside or bongkrekic acid, inhibitor of adenine nucleotide translocator (ANT), affects MPT, ANT was thought to be required for MPT^20^. However, mitochondria from ANT-deficient mice reveal that ANT is not essential for MPT induction but is involved in Ca^2+^ sensitivity of MPT^21^. Ro68-3400 was identified by a screening of small molecule compounds, and it potently inhibits mitochondrial swelling. The compound was demonstrated to bind to voltage-dependent anion channel (VDAC), suggesting VDAC is a potential mediator of MPT^22^. However, VDAC was shown to be dispensable to MPT by a loss of function study of VDAC^23^. PPIF, the target of CsA in the mitochondrial matrix, is the most well-known mediator of MPT. Actually, mitochondria from PPIF-deficient mice have been demonstrated to show resistance to swelling induced by Ca^2+^ ^24, 25^. In addition, recent studies identified several inhibitors of MPT^26–29^. These compounds seem to be valuable chemical biological tools to understanding mechanism of MPT, but molecular mechanisms of their inhibitory actions are completely unknown.

In the present study, we screened small molecule compounds for MPT inhibitors by using isolated mitochondria. We identified DS44170716 as showing a significant protective effect against Ca^2+^-induced mitochondrial swelling, and found its action to be independent of PPIF. The compound protected human liver cultured cells from Ca^2+^ ionophore-induced death with a level of protection similar to CsA. Further analysis revealed that DS44170716 potently blocks Ca^2+^ overload into mitochondria by inhibiting mitochondrial complex III, IV and V. The study shows that the blockade of Ca^2+^ influx by inhibition of mitochondrial complex activities is a novel mechanism for protection against Ca^2+^-induced cell death.

## Results

### DS44170716 is a novel inhibitor of mitochondrial permeability transition

**Figure 1:**
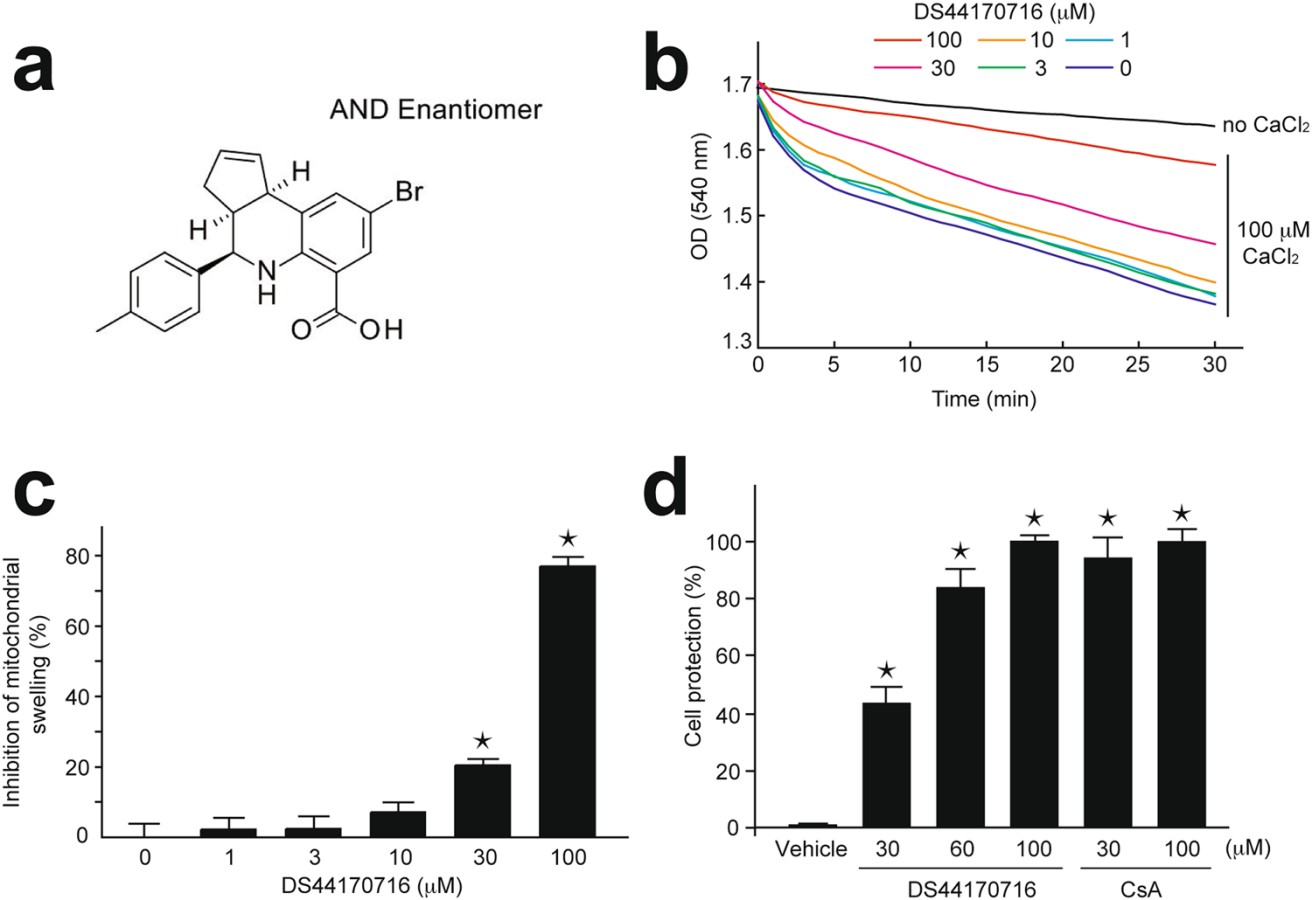
DS44170716 inhibits mitochondrial permeability transition. (**a**) Structure of DS44170716. (**b**) Effect of DS44170716 on mitochondrial swelling. Representative temporal profiles are shown. (**c**) Inhibition rate of mitochondrial swelling by DS44170716. The levels of OD (540 nm) 30 min after application of CaCl_2_ were used for the inhibition rate. Inhibition 0% or 100% was defined as CaCl_2_-treated vehicle data or no CaCl_2_ data, respectively. Data obtained from 3 independent samples are shown as the mean with SEM. (**d**) Effects of DS44170716 on Ca^2+^-induced death in HepG2 cells. Cell death levels were measured by LDH released from the cells. Cell protection 0% or 100% was defined as the mean of vehicle data or mean of 100 μM CsA, respectively. Raw data are presented in Supplemental Fig. 1. Data obtained from 4 independent samples are shown as the mean with SEM. Asterisk shows P < 0.05 compared to vehicle group (Dunnett’s test).

First, we screened synthetic small-molecule compounds for novel inhibitors of MPT. MPT is detectable as mitochondrial swelling, and inhibitors of the swelling are referred to as MPT inhibitor^4, 6, 19, 20, 26–29^. In the present study, freshly-isolated mitochondria from rat liver were used for the swelling assay. Application of 100 μM Ca^2+^ to mitochondrial solution resulted in a temporal decrease in absorbance at 540 nm. We identified a compound DS44170716 that dose-dependently inhibited decrease of absorbance in a range of 1 μM to 100 μM (Fig. 1a–c). This result suggested that DS44170716 is a novel inhibitor of MPT in the rat liver mitochondria.

Then, we analyzed the effect of DS44170716 on Ca^2+^-induced cell death. In order to evoke Ca^2+^ overload into mitochondria, human liver cell line HepG2 cells were stimulated by 10 μM A23187 for 3 hours. To evaluate cell protective effects, CsA was incubated with the calcium ionophore. Cell death was then evaluated by measuring released lactate dehydrogenase (LDH) within the supernatant of the cell culture. The CsA treatment protected cells from Ca^2+^-induced cell death (Fig. 1d and Supplemental Fig. 1). Similarly, DS44170716 blocked the Ca^2+^-induced cell death in a dose-dependent manner. When compared to the effect of CsA, the protective effect of 100 μM DS44170716 was similar in extent to that of 100 μM CsA. These results showed that DS44170716 has a potent protective effect against Ca^2+^-induced death in human liver cells.

### DS44170716 blocks MPT in a PPIF-independent manner

**Figure 2:**
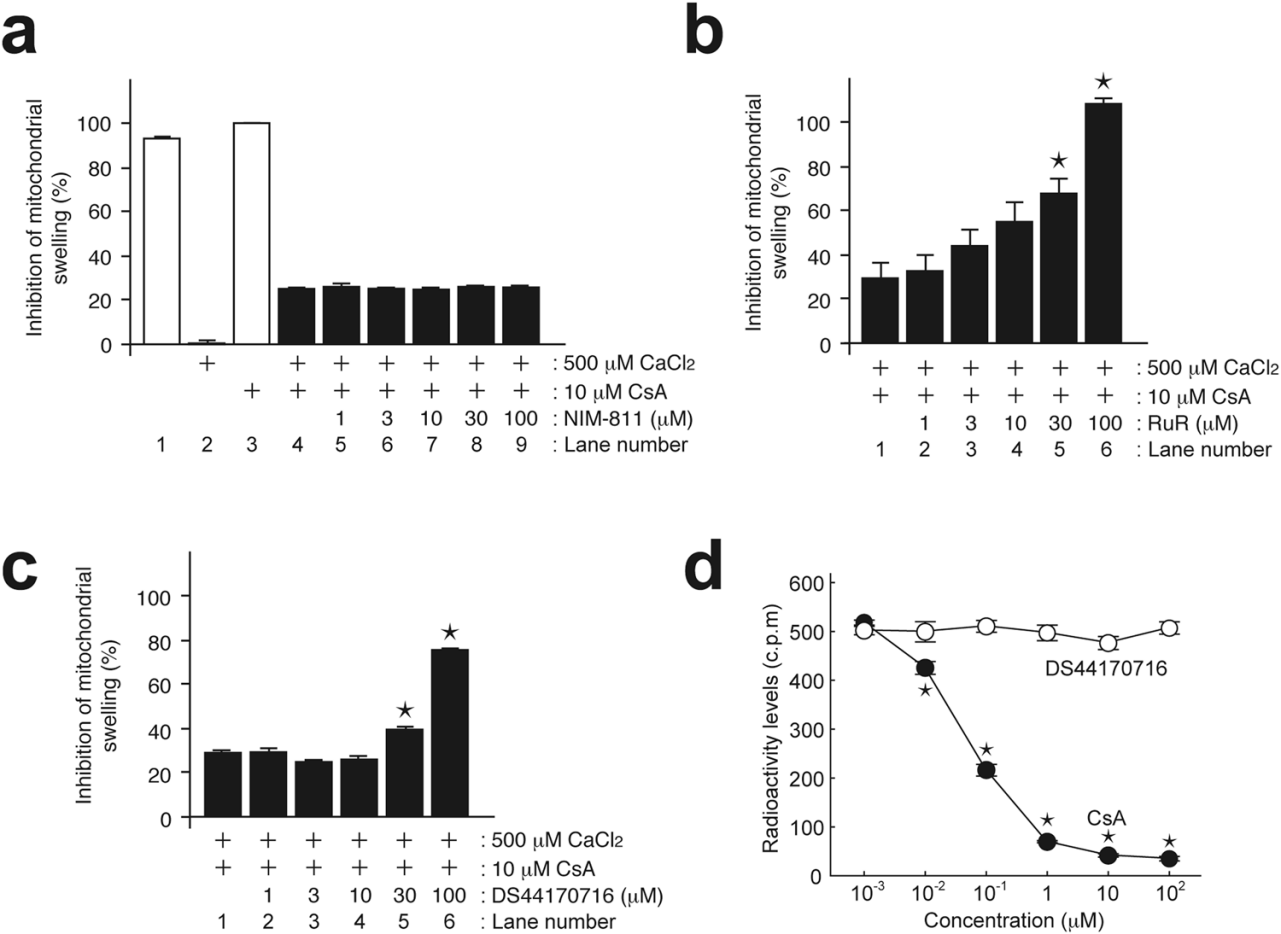
DS44170716 inhibits mitochondrial swelling in a different mechanism of cyclosporin A. (**a**) Effect of NIM-811 on swelling in the presence of CsA. (**b**) Effect of ruthenium red on swelling in the presence of CsA. (**c**) Effect of DS44170716 on swelling in the presence of CsA. Inhibition 0% or 100% was defined as 500 μM CaCl_2_-treated mitochondria (**a**, Lane 2) or 10 μM CsA-treated mitochondria (**a**, Lane 3), respectively. Data obtained from 3 independent samples are shown as the mean with SEM. Raw data are presented in Supplemental Fig. 2. (**d**) No significant binding activity of DS44170716 to cyclosporin A-binding site of PPIF. Binding activities of radio-labeled CsA to PPIF were measured by SPA assay. Data obtained from 3 independent samples are shown as the mean with SEM. Asterisk shows P < 0.05 compared to vehicle group (Dunnett’s test).

We then investigated the action mechanism of DS44170716. First, we determined whether or not DS44170716 depended on the CsA-target molecule PPIF to exert its inhibitory effect. A previous study clearly determined PPIF dependency by an assay using mitochondria without PPIF activity^13^. In order to develop swelling assay without PPIF activity, we used a high dose of CaCl_2_ (500 μM) for strong induction of mitochondrial swelling (Supplemental Fig. 2 and Fig. 2a, compare Lane 1 with Lane 2). The swelling was partially blocked by 10 μM CsA (Fig. 2a, compare Lane 3 with Lane 4). In this condition, PPIF activity was completely inactivated, because additive effects by high dose of CsA (100 μM) were not observed (data not shown). We then checked whether additional treatment of another drug added inhibitory effects against the swelling. NIM-811, an analog of CsA that inhibits mitochondrial PPIF, showed no additional effect on the level of mitochondrial swelling (Fig. 2a, compare Lane 4 with Lanes 5 to 9). This suggests that drugs targeting PPIF would show no additional protection in this assay. Next we tested Ruthenium red (RuR), a potent blocker of the mitochondrial calcium uniporter (MCU), which is an inward-rectifying, highly-selective Ca^2+^ channel in the mitochondrial inner membrane^1, 3^. Interestingly, RuR showed an additional protective effect against the mitochondrial swelling in a dose-dependent manner (Fig. 2b, compare Lane 1 with Lanes 2 to 6). These results indicated the assay as being useful for classifying inhibitors into PPIF-dependent inhibitors (like NIM-811) or PPIF-independent inhibitors (like RuR). When DS44170716 was applied in this assay, it showed an additional protective effect against mitochondrial swelling in a dose-dependent manner (Fig. 2c, compare Lane 1 with Lanes 2 to 6). These results are not co-effect of CsA, because protection of the mitochondrial swelling was observed in single treatment of DS44170716 (Fig. 1), NIM-811 or RuR (data not shown)^30, 31^. Therefore, we concluded that the inhibitory mechanism of DS44170716 is clearly different from that of CsA.

We further investigated the PPIF-dependency of DS44170716 by evaluating binding activity of DS44170716 to PPIF protein. PPIF stably binds to cyclosporin A, and the binding region of PPIF is important for peptydil-proryl isomerase activity^3, 4^. We established a scintillation proximity assay (SPA) to evaluate binding activity of the compounds to PPIF. After incubation of radio-labeled CsA with PPIF, the binding levels were quantified by using SPA beads following immunoprecipitation with an anti-PPIF antibody. Binding of radio-labeled CsA to PPIF was competitively inhibited by preincubation of non-labeled CsA in a dose-dependent manner (Fig. 2d, black circles). On the other hand, DS44170716 showed no effects on binding of radiolabelled CsA to PPIF (Fig. 2d, white circles). These results suggest that DS44170716 shows no binding activities at the CsA-binding site on the PPIF protein.

### DS44170716 inhibits mitochondrial Ca^2+^ influx

**Figure 3:**
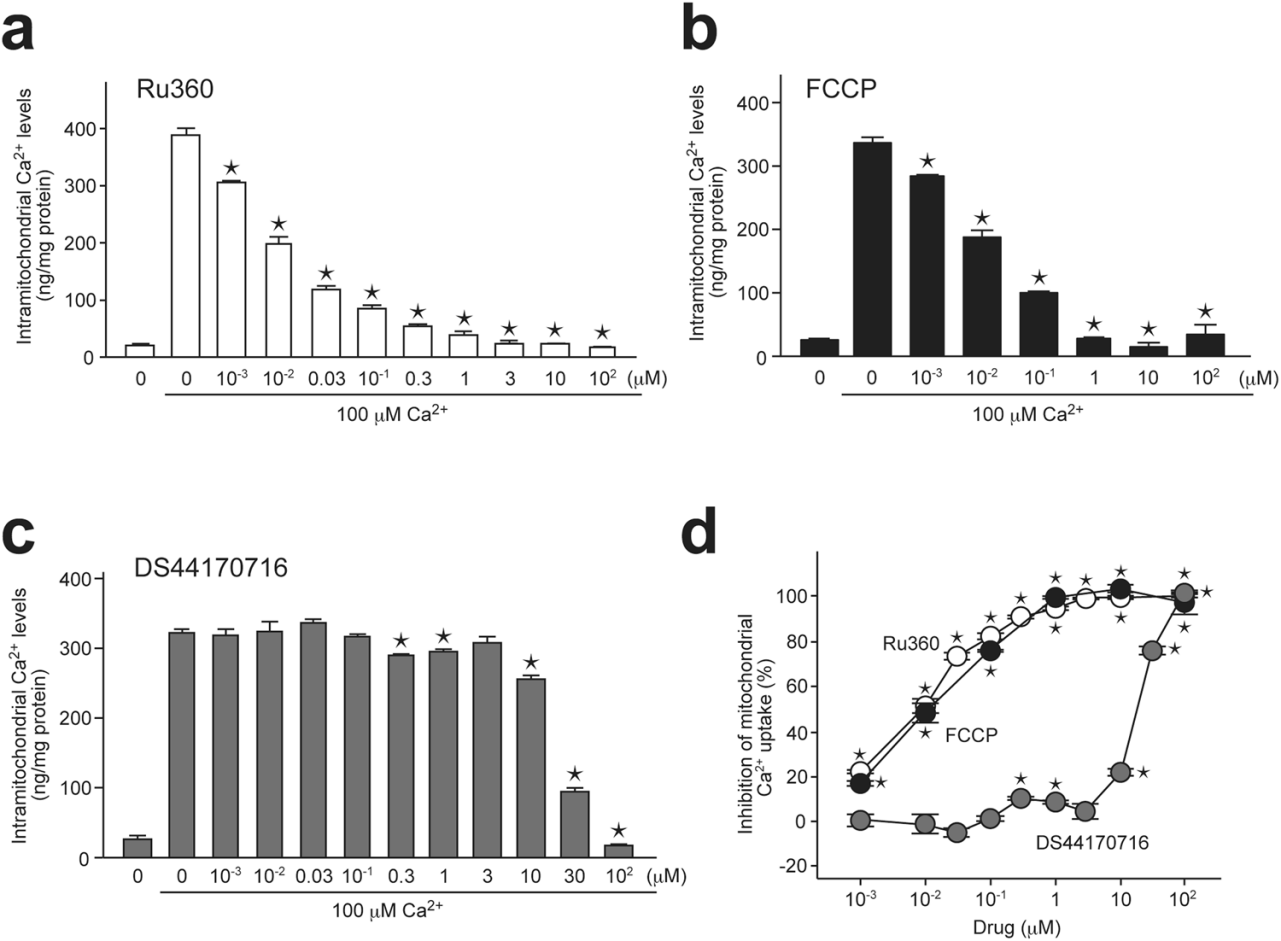
DS44170716 inhibits mitochondrial Ca^2+^ uptake in isolated mitochondria from pig heart. (**a**) Effect of Ru360 on Ca^2+^ uptake activities of isolated mitochondria. (**b**) Effect of FCCP on Ca^2+^ uptake activities of isolated mitochondria. (**c**) Effect of DS44170716 on Ca^2+^ uptake activities of isolated mitochondria. (**d**) Inhibitory effects of drugs on Ca^2+^ uptake activities of isolated mitochondria. Inhibition 100 or 0% was defined as the mean of vehicle-treated samples or 100 μM CaCl_2_-treated samples, respectively. Data obtained from 3 independent samples are shown as the mean with SEM. Asterisk shows P < 0.05 compared to vehicle group (Dunnett’s test).

Next we investigated effects of DS44170716 on dynamics of Ca^2+^ uptake into mitochondria. Because Ca^2+^ blockade is a possible mechanism for inhibition of swelling (Fig. 2b), an atomic absorbance spectrometer-based method was developed for detection of Ca^2+^ uptake activities. First, we developed a Ca^2+^ uptake assay by using rat mitochondria from the liver and heart. Significant inhibitory effects of DS44170716 were detected in the assay, but the signal to noise ratio was low (data not shown). Then we used fresh, isolated mitochondria from pig heart. The mitochondria were incubated with 100 μM Ca^2+^, and then intramitochondrial Ca^2+^ levels were detected. We detected a significant increase of Ca^2+^ levels in the mitochondria (Fig. 3a). The increase was dose-dependently blocked by pretreatment of the MCU blocker Ruthenium 360 (Ru360) (Fig. 3a). These results show that the significant increase of Ca^2+^ was mediated by the MCU. We then checked the other inhibitory mechanism by using an uncoupler of mitochondrial membrane potential. The driving force of mitochondrial Ca^2+^ uptake is a proton gradient across the inner membrane produced by oxidative phosphorylation^3^. In the assay, carbonyl cyanide-p-trifluoromethoxyphenylhydrazone (FCCP) also blocked accumulation of mitochondrial Ca^2+^ in a dose-dependent manner (Fig. 3b), which was consistent with a previous study^32^. Importantly, we found that DS44170716 blocked accumulation of mitochondrial Ca^2+^ in a dose-dependent manner (Fig. 3c). Inhibition rates of the compounds were analyzed as depicted in Fig. 3d. These results showed that DS44170716 potently blocks Ca^2+^ influx into mitochondria, the initial event of Ca^2+^-induced MPT^33^.

**Figure 4:**
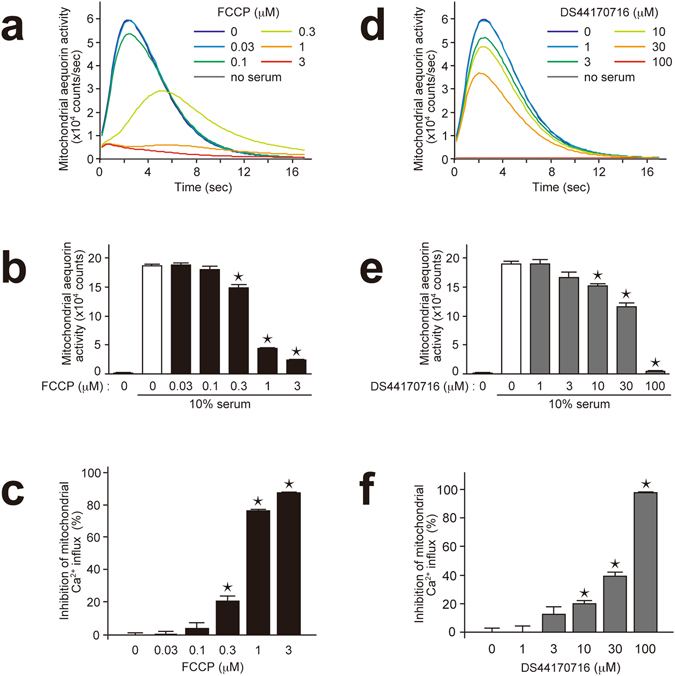
DS44170716 inhibits serum-induced mitochondrial Ca^2+^ influx in human HEK293A cells. (**a**) Representative temporal profiles of FCCP effect on serum-induced mitochondrial Ca^2+^ influx in human HEK 293 A cells. (**b**) FCCP effect on serum-induced mitochondrial Ca^2+^ influx in human HEK 293 A cells. The data shown were total mitochondrial Ca^2+^ influx from 0 to 17 seconds after the serum treatment. (**c**) Inhibitory effect of FCCP on serum-induced mitochondrial Ca^2+^ influx in human HEK 293 A cells. (**d**) Representative temporal profiles of DS44170716 effect on serum-induced mitochondrial Ca^2+^ influx in human HEK 293 A cells. (**e**) DS44170716 effect on serum-induced mitochondrial Ca^2+^ influx in human HEK 293 A cells. (**f**) Inhibitory effect of DS44170716 on serum-induced mitochondrial Ca^2+^ influx in human HEK 293 A cells. Data obtained from 3 independent samples are shown as the mean with SEM. Asterisk shows P < 0.05 compared to vehicle group (Dunnett’s test).

The inhibitory effect against the mitochondrial Ca^2+^ influx was also investigated by using a cultured cell assay. In order to evaluate mitochondrial Ca^2+^ influx in intact cells, we established HEK293A cell lines stably expressing Ca^2+^ indicator protein aequorin in the mitochondria. Treatment of 10% fetal bovine serum induced an acute increase of mitochondrial Ca^2+^ influx (Fig. 4a). In this assay, the positive control FCCP abolished the serum-induced increase of mitochondrial Ca^2+^ (Fig. 4a–c). Similarly, DS44170716 blocked the mitochondrial Ca^2+^ influx in a dose-dependent manner (Fig. 4d–f). These results indicated that DS44170716 inhibits mitochondrial Ca^2+^ influx in human living cells.

### DS44170716 decreases mitochondrial membrane potential

**Figure 5:**
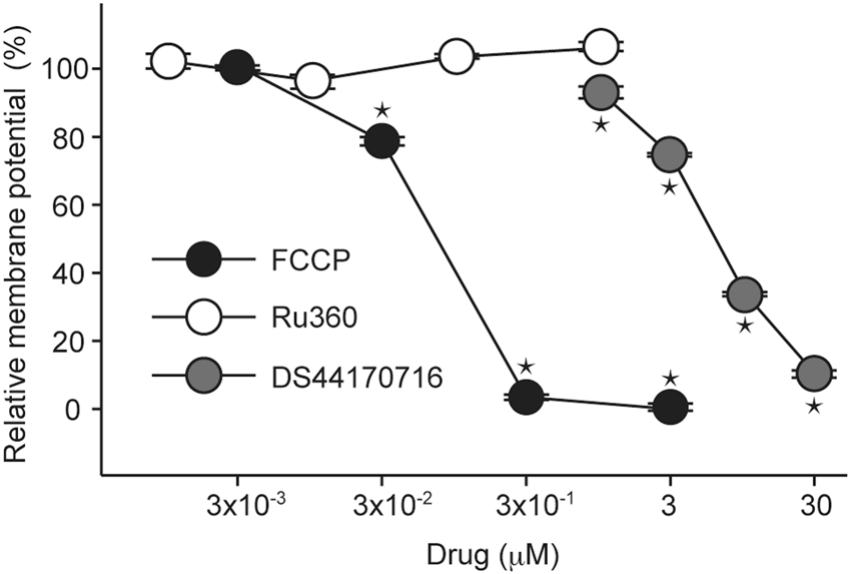
DS44170716 decreases mitochondrial membrane potential in isolated mitochondria from pig heart. Relative membrane potential 0% or 100% was defined as 3 μM FCCP data or vehicle data, respectively. Data obtained from 4 independent samples are shown as the mean with SEM. Asterisk shows P < 0.05 compared to vehicle group (Dunnett’s test).

We next investigated the inhibitory mechanism of mitochondrial Ca^2+^ influx by DS44170716. As shown in Fig. 3, inhibition of Ca^2+^ uptake is caused by two mechanisms: blockade of the MCU or disruption of the membrane potential. We developed a mitochondrial membrane potential assay by using a fluorescence chemical probe JC-1 (Fig. 5). In the assay, positive control FCCP significantly disrupted the mitochondrial membrane potential (Fig. 5, black circles). On the other hand, MCU blocker Ru360 had no effect on the membrane potential (Fig. 5, white circles). In the assay, DS44170716 decreased membrane potential in a dose-dependent manner (Fig. 5, gray circles). These results indicated that the blocking of Ca^2+^ entry by DS44170716 is mediated by the reducing effect on the membrane potential.

**Figure 6:**
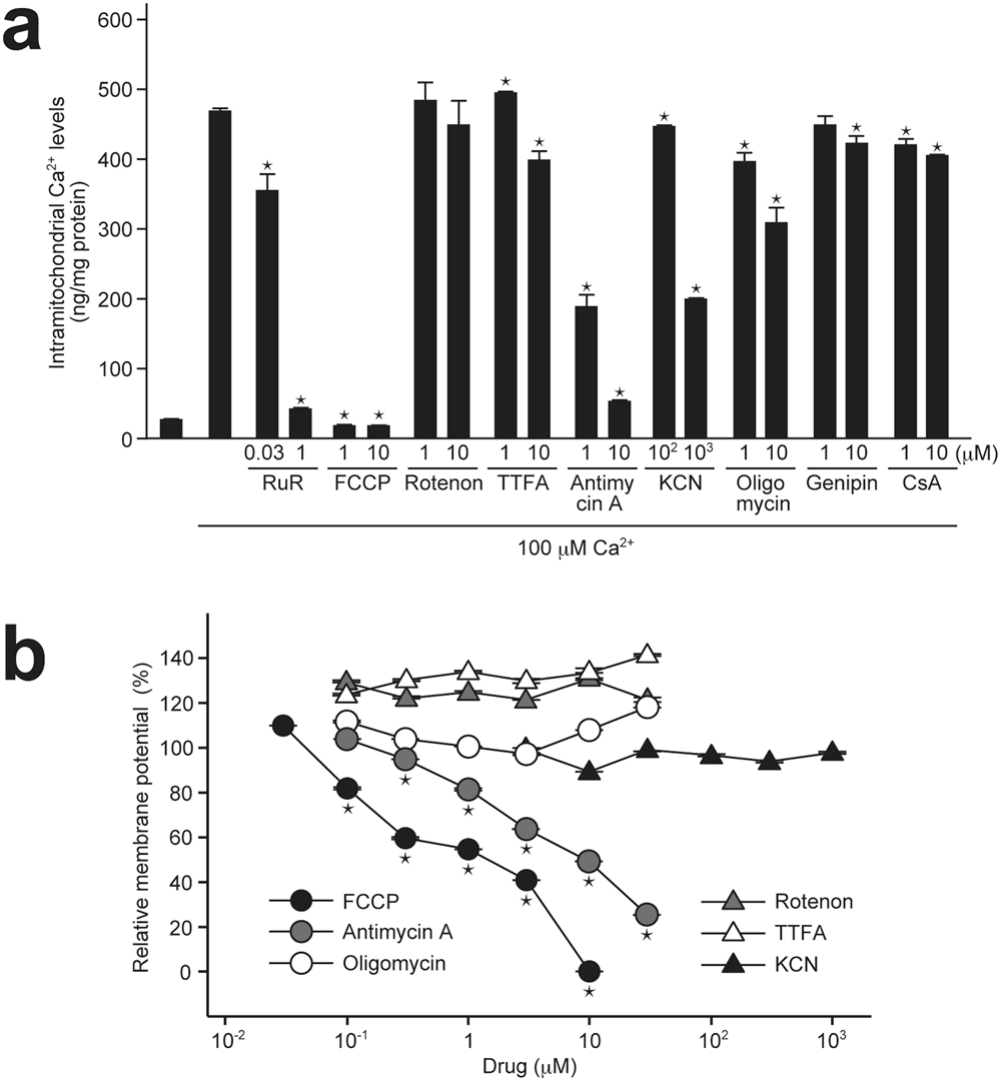
Screening of mitochondrial drugs affecting Ca^2+^ uptake and membrane potential. (**a**) Effect of mitochondrial drugs on Ca^2+^ uptake activities in isolated mitochondria from pig heart. Data obtained from 3 independent samples are shown as the mean with SEM. Asterisk shows P < 0.05 compared to vehicle group (Dunnett’s test). (**b**) Effect of mitochondrial drugs on membrane potential in isolated mitochondria from pig heart. Data obtained from 4 independent samples are shown as the mean with SEM. Asterisk shows P < 0.05 compared to minimum dose group (Dunnett’s test).

In general, uncouplers of mitochondrial membrane potential, such as FCCP, are used to induce mitochondrial swelling^3, 4, 19^. Because DS44170716 protected cells from mitochondrial swelling (Fig. 1), we hypothesized that DS44170716 was not an uncoupler agent, but specifically inhibited mitochondrial enzyme(s) to decrease the membrane potential. In order to understand what kinds of drugs influence mitochondrial Ca^2+^ uptake activities, we analyzed the effects of a series of mitochondrial drugs by the Ca^2+^ uptake assay. The positive controls RuR and FCCP inhibited the Ca^2+^ uptake activities, whereas the complex I inhibitor rotenone showed no significant inhibition (Fig. 6a). Complex II inhibitor 2-thenoyltrifluoroacetone (TTFA) showed weak inhibitory effect against the Ca^2+^ uptake activities. On the other hand, complex III inhibitor antimycin A strongly inhibited the Ca^2+^ uptake activities in a dose-dependent manner. A high dose of complex IV inhibitor KCN or complex V inhibitor oligomycin showed an inhibitory effect against the Ca^2+^ uptake activities. A small effect was observed by the treatment with genipin, an inhibitor of uncoupling protein 2^34^, or CsA (Fig. 6a). These results suggested that inhibition of complex III, IV or V gives rise to the reduction of mitochondrial Ca^2+^ uptake.

Next we tested effects of the mitochondrial drugs on the membrane potential. While positive control FCCP reduced the potential (Fig. 6b, black circles), rotenone, TTFA, KCN and oligomycin showed no reducing effect on the membrane potential (Fig. 6b, gray triangles, white triangles, black triangles and white circles, respectively). We found that antimycin A strongly reduced the potential in a dose-dependent manner (Fig. 6b, gray circles). These results suggested that inhibition of mitochondrial Ca^2+^ uptake by complex III inhibitors (Fig. 6a) is mediated by a strong reducing action against the mitochondrial membrane potential.

### DS44170716 inhibits enzyme activities of respiratory chain complexes

**Figure 7:**
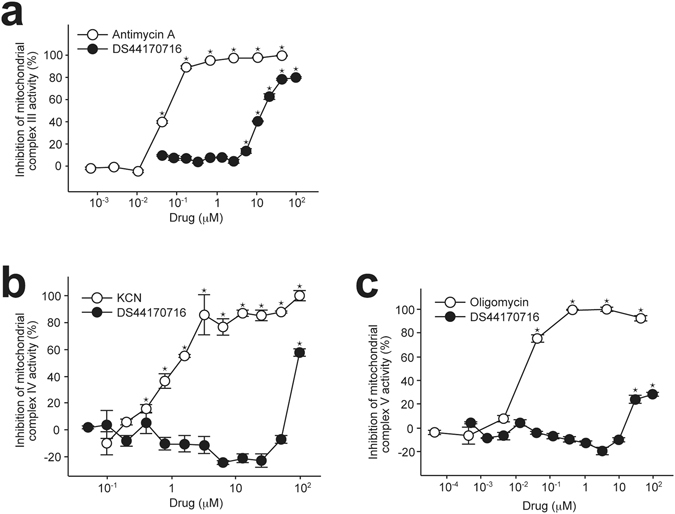
DS44170716 inhibits enzyme activities of respiratory chain complexes. (**a**) Effect of DS44170716 on enzyme activities of mitochondrial complex III. (**b**) Effect of DS44170716 on enzyme activities of mitochondrial complex IV. (**c**) Effect of DS44170716 on enzyme activities of mitochondrial complex V. Inhibition 0% or 100% was defined as the mean of vehicle-treated samples or samples that were maximally inhibited by the positive control. Data obtained from 3 independent samples are shown as the mean with SEM. Asterisk shows P < 0.05 compared to vehicle group (Dunnett’s test).

Finally, we tested the effect of DS44170716 on enzyme activities of mitochondrial complexes. The mitochondrial drug screening revealed that inhibition of complex III, IV or V results in the inhibition of Ca^2+^ uptake (Fig. 6a). Based on this observation, we tested the effect of DS44170716 on enzyme activities of complex III, IV and V. In the analysis of complex III enzyme activities, positive control antimycin A dose-dependently inhibited the activities (Fig. 7a, white circles). In the assay, DS44170716 also inhibited complex III activities in a dose-dependent manner (Fig. 7a, black circles). Next, effects of the compound on complex IV enzyme activities were analyzed. We confirmed that positive control KCN inhibited the activities (Fig. 7b, white circles). In this condition, only 100 μM DS44170716 showed strong inhibition of the activities (Fig. 7b, black circles). Finally, we analyzed the action of DS44170716 on complex V activities. The positive control oligomycin inhibited the activities in a dose-dependent manner (Fig. 7c, white circles). In the assay, 30 or 100 μM DS44170716 showed inhibitory action against the complex V activities. These results implied that the blockade of mitochondrial Ca^2+^ uptake by DS44170716 is mediated by its inhibitory actions against mitochondrial complex III, IV and V.

## Discussion

In the present study, we found that DS44170716 potently blocks MPT in a PPIF-independent manner. Importantly, DS44170716 protects human HepG2 cells from Ca^2+^-induced death with an efficacy similar to that of CsA. This inhibition is mediated by blockade of Ca^2+^ influx into the mitochondria. DS44170716 reduces mitochondrial membrane potential and inhibits enzyme activities of complex III, IV and V. These findings of DS44170716 provide a novel viewpoint in blocking mechanism of MPT for protection of cells from Ca^2+^-induced injury.

**Figure 8:**
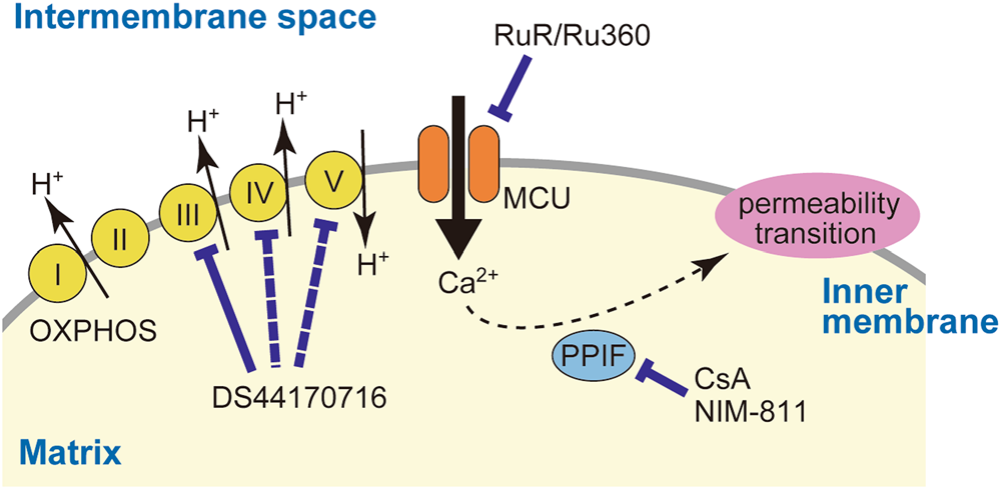
Action mechanism of DS44170716. Mitochondrial respiratory chain complexes generate proton gradient, which is a driving force for Ca^2+^ uptake. Ca^2+^ overload into the matrix induces mitochondrial permeability transition (MPT). DS44170716 strongly inhibits complex III and weakly inhibits complex IV and V to decrease the potential. The decrease inhibits mitochondrial Ca^2+^ uptake and MPT. RuR or Ru360 blocks Ca^2+^ uptake by acting on MCU. CsA or NIM-811 inhibits PPIF, a central player promoting MPT.

There are several reports proposing novel models of mPTP^35–39^. Interestingly, complex V is thought to be plausible component of the pore^39^, and inhibitors of mitochondrial respiratory chain complexes block MPT^40–42^. Because DS44170716 inhibits enzyme activities of complex III, IV and V, the triple inhibitory actions may be important for the strong inhibitory effect on Ca^2+^ uptake and MPT (Fig. 8). In addition, the present analysis could not exclude a possibility that DS44170716 directly inhibits activity of MCU protein complex. Further studies are important to elucidate action mechanism of DS44170716 in detail.

In drug development research, the regulation of MPT is thought to be a promising target for tissue protection. CsA and its non-immunosuppressive analogs have been shown to protect from reperfusion injury in several animal studies^6–12^. In a phase 2 clinical study, effects of cyclosporin were investigated in 58 patients with acute ST-elevation myocardial infarction^43^. The patients who received cyclosporin immediately before undergoing percutaneous coronary intervention (PCI) showed a smaller infarct than that seen in those receiving the placebo. On the other hand, the phase 3 clinical study using 970 patients revealed that CsA showed no significant effect on the reperfusion injury after PCI^44^. As discussed in the reports, an inhibitor specific to MPT may be important for clinical efficacy because CsA has nonmitochondrial effects including potent immunosuppressive activity. Therefore, a PPIF-independent target is one of the next candidates for development of MPT inhibitors^26–29^.

Recent molecular biological studies have identified components of the mitochondrial calcium uniporter^45–51^. In addition, a human genetic study showed that a mutation of *MICU1*, the gene encoding a regulatory subunit of the uniporter, causes an increase of mitochondrial Ca^2+^ influx and promotes the development of brain and muscle disorders^52^. These studies suggest that blocking the mitochondrial Ca^2+^ entry is a promising approach to protecting tissue from injury. The present study identifies a novel MPT inhibitor that blocks mitochondrial Ca^2+^ uptake by interfering with the enzyme activities of mitochondrial complexes. Because DS44170716 directly inhibits respiratory chains, the compound may have safety problem in the respect of drug development. For the purpose, a direct inhibitor of the MCU seems to be hopeful as a next mitochondria-targeting drug. Recently, we found a cell-permeable blocker of the uniporter by using a novel screening method (in preparation for publication). Blockade of Ca^2+^ overload is an emerging strategy for drug development seeking to satisfy the unmet medical needs of mitochondrial diseases.

## Methods

### Animals and reagents

Nine-week-old male Wistar rats were purchased from Japan SLC (Hamamatsu, Japan). All experimental procedures were performed in accordance with the in-house guidelines of the Institutional Animal Care and Use Committee of Daiichi-Sankyo, which is an accredited organization by Association for Assessment and Accreditation of Laboratory Animal Care (AAALAC). The experimental protocols for the present study were approved by the in-house committee (protocol application number A1400451). Every effort was made to minimize animal suffering and to reduce the number of animals employed. All animal studies were also conducted in accordance with the ARRIVE guidelines^53, 54^. The following were also purchased: fresh pig hearts from Tokyo Shibaura Zouki Co., Ltd. (Tokyo, Japan); pIRES-puro vector from Clontech Laboratories, Inc. (CA, USA); HEK293A cells, pcDNA3.1, Hanks Balanced Salt Solution, coelenterazine h and mitochondria isolation kit from Thermo Fisher Scientific Inc. (MA, USA); MitoTox OXPHOS Complex III Activity Kit, Complex IV Human Enzyme Activity Microplate Assay Kit and MitoTox Complex V OXPHOS Activity Microplate Assay from Abcam Plc (Cambridge, UK); fetal bovine serum and ruthenium red, rotenone, antimycin A, KCN, oligomycin, FCCP, genipin and cyclosporin A from Sigma-Aldrich, Inc. (MO, USA); JC-10 from Enzo Life Sciences, Inc. (NY, USA); 2-Thenoyltrifluoroacetone (TTFA) from Caymen Chemical (MI, USA); DS44170716 from specs (Zoetermeer, The Netherlands). NIM-811 was synthesized as described previously^55^.

### Isolation of mitochondria

Mitochondria were isolated from rat livers or pig hearts through sequential centrifugation by using a mitochondria isolation kit for tissue (Thermo Scientific, cat. #89801). To obtain mitochondria, tissues were disrupted with a glass Teflon homogenizer. Nuclei and plasma membrane fractions were separated first by mild centrifugation (700 *g*, 10 min), and mitochondria were then spinned down at a higher speed (3,000 *g*, 20 min). All procedures were carried out at 4 °C.

### Mitochondrial swelling assay

Swelling of mitochondria was detected as described previously^22^. Rat liver mitochondria were suspended in a swelling assay buffer (150 mM sucrose, 50 mM KCl, 2 mM KH_2_PO_4_, 5 mM succinic acid, 20 mM Tris-HCl [pH7.4]). Five minutes after application of DS44170716, 100 μM CaCl_2_ was added and OD (540 nm) was measured per minute for 30 min by using a SpectraMax Plus (Molecular Devices). In order to test PPIF-dependency, NIM-811, RuR or DS44170716 was applied in the mitochondrial suspension with 10 μM CsA, and then 500 μM CaCl_2_ was applied.

### PPIF-CsA binding assay

Cyclosporin A or DS44170716 was incubated with 100 nM human PPIF protein (ATGen, cat. #PPF0901) in binding buffer (PBS [Sigma-Aldrich, cat. #D8662] containing 0.05% tween and 0.1% BSA) for 3 h at room temperature. Then, 100 nM tritium-labeled cyclosporin A (PerkinElmer, cat. #NET1159001MC) was added and incubated for 1 h. To immunoprecipitate PPIF protein, 20 ng of anti-PPIF antibody (ATGen, cat. #ATGA0139) was added and incubated for 1 h. SPA beads (PerkinElmer, cat. #RPN141) were added and incubated for 20 min, then the radioactivity levels were measured with a TopCount® (PerkinElmer, Inc.).

### Ca^2+^ uptake assay using isolated heart mitochondria

Mitochondria were isolated by using the mitochondria isolation kit and then dissolved in the swelling buffer. Thirty minutes after application of CaCl_2_ (final concentration of 100 μM) to the solution, the mitochondria were collected by centrifugation (3,000 *g*) at 4 °C. The pellets were resuspended in the swelling buffer containing 1 μM ruthenium red. After collection of the mitochondria by centrifugation (3,000 *g*), the pellets were dried and dissolved by 40 μL of sulfuric acid at 95 °C. The solution was then diluted by water, and Ca^2+^ concentration of the solution was measured by atomic absorbance spectrometer (Hitachi High-Technologies Corporation, Z-2710).

### Mitochondrial membrane potential assay

Pig heart mitochondria were prepared as described above and suspended in the swelling buffer containing 1 μM JC-10. The protein concentration of the solution was 5 mg/mL. Five minutes after incubation of the mitochondria with DS44170716 at room temperature, fluorescence intensities were measured by a FlexStation® 3 (Molecular Devices, LLC.) using the green channel (excitation/emission wavelength: 485 nm/538 nm) or the red channel (excitation/emission wavelength: 485 nm/612 nm).

### Aequorin assay

For dynamic measurements of mitochondrial Ca^2+^ levels in intact cells, HEK293A cells were stably transfected with pIRES-puro vector expressing mitochondria-targeted aequorin^32^. One day after plating on a 15-cm dish (8 × 10^6^ cells/dish), the cells were harvested and incubated with 2.5 μM coelenterazine h in aequorin assay buffer (200 mM Hanks Balanced Salt Solution, 25 mM HEPES [pH 7.0] and 0.1% bovine serum albumin) for 2 hours at room temperature. The cells (8.1 × 10^4^ cells/well) were then treated with DS44170716 for 20 min in a 96-well plate at room temperature. For induction of intracellular Ca^2+^, the cells were treated with 10% fetal bovine serum, and luminescence levels were measured by using a Centro LB960 luminometer (Berthold Technologies USA).

### LDH assay for cell death measurement

One day after plating of HepG2 cells (5 × 10^4^ cells/well) in a 96-well dish with DMEM (Invitrogen, cat. #11965), cells were treated with medium (Invitrogen, Opti-MEM I Reduced Serum Medium, cat. #31985) containing 10 μM A23187 and drugs for 3 hours. Then, supernatant was collected and LDH activities were measured by a Cytotoxicity Detection Kit (LDH) (Roche, cat #11644793001).

### Statistics for experimental data

For multiple comparisons of experimental data, Dunnett’s test was performed by using web-based statistical analysis tools (http://www.gen-info.osaka-u.ac.jp/MEPHAS/). The data presented in the Figures are mean with SEM of 3 or 4 technical replicates as described in the legends. Similar results were obtained in at least 3 independent biological replicates.

## Electronic supplementary material

Supplemental Information
