# Supplementary material for: A small-molecule DS44170716 inhibits Ca2+-induced mitochondrial permeability transition
Source: Sci Rep. 2017 Jun 20;7:3864. doi: 10.1038/s41598-017-03651-7 (PMC5478606; doi:10.1038/s41598-017-03651-7)
Supplement: Supplementary file 1 — Supplemental Information [file 41598_2017_3651_MOESM1_ESM.pdf]

A small-molecule DS44170716 inhibits Ca^{2+}-induced mitochondrial permeability transition

Naohiro Kon[1]*, Atsushi Satoh[2] and Naoki Miyoshi[3]

[1]Medical Science Department, Daiichi Sankyo Co., Ltd., Tokyo, Japan

[2]Manufacturing Department III, Kitasato Daiichi Sankyo Vaccine Co., Ltd., Saitama, Japan

[3]End-Organ Disease Laboratories, Daiichi Sankyo Co., Ltd., Tokyo, Japan

*konntgf@gmail.com

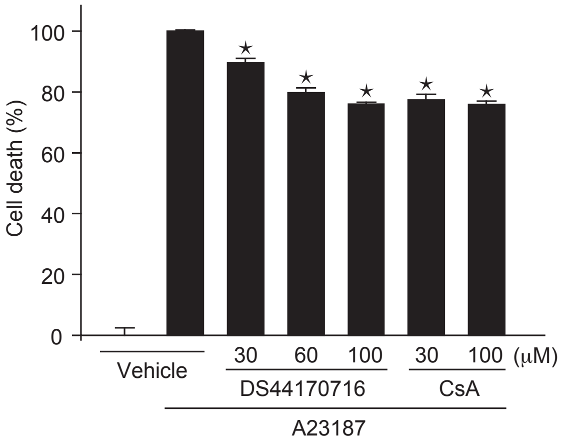

Supplemental Figure 1

Effects of DS44170716 on Ca^{2+}-induced death in HepG2 cells.

Cell death 100% or 0% was defined as the mean of vehicle-treated data with or without A23187, respectively. Data obtained from 4 independent samples are shown as the mean with SEM. Asterisk shows $P < 0.05$ compared to vehicle group (Dunnett's test).

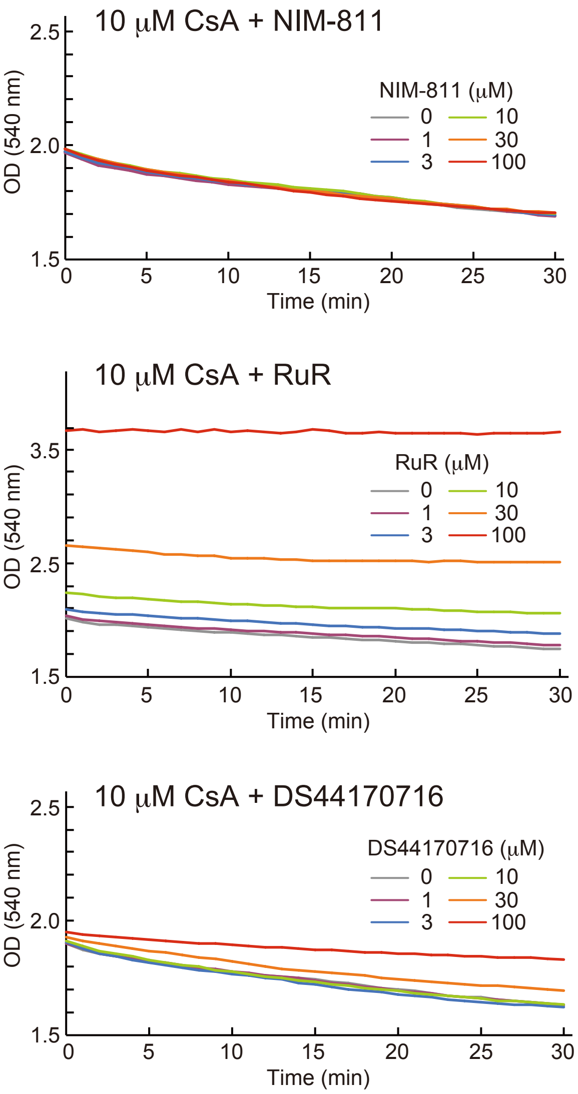

Supplemental Figure 2
Effects of MPT inhibitors on mitochondrial swelling in the presence of CsA.
At time 0, 500 μM $CaCl_2$ was applied to mitochondria from rat liver in the presence of CsA. NIM-811, RuR or DS44170716 was additionally applied in each sample, and temporal changes of OD (540 nm) were measured. By using the raw data, inhibition of mitochondrial swelling (%) was calculated (Fig. 2a-c). In each graph, OD level of 30 min was subtracted from that of 0 min for calculation of swelling level. The calculation method is useful especially in the case that OD levels were strongly affected by colored drug, such as RuR.
